# Meta-Analysis of Parkinson's Disease Transcriptome Data Using TRAM Software: Whole Substantia Nigra Tissue and Single Dopamine Neuron Differential Gene Expression

**DOI:** 10.1371/journal.pone.0161567

**Published:** 2016-09-09

**Authors:** Elisa Mariani, Flavia Frabetti, Andrea Tarozzi, Maria Chiara Pelleri, Fabrizio Pizzetti, Raffaella Casadei

**Affiliations:** 1 Department for Life Quality Studies, University of Bologna, Rimini, Italy; 2 Department of Experimental, Diagnostic and Specialty Medicine, University of Bologna, Bologna, Italy; Duke University, UNITED STATES

## Abstract

The understanding of the genetic basis of the Parkinson's disease (PD) and the correlation between genotype and phenotype has revolutionized our knowledge about the pathogenetic mechanisms of neurodegeneration, opening up exciting new therapeutic and neuroprotective perspectives. Genomic knowledge of PD is still in its early stages and can provide a good start for studies of the molecular mechanisms that underlie the gene expression variations and the epigenetic mechanisms that may contribute to the complex and characteristic phenotype of PD. In this study we used the software TRAM (Transcriptome Mapper) to analyse publicly available microarray data of a total of 151 PD patients and 130 healthy controls substantia nigra (SN) samples, to identify chromosomal segments and gene loci differential expression. In particular, we separately analyzed PD patients and controls data from post-mortem snap-frozen SN whole tissue and from laser microdissected midbrain dopamine (DA) neurons, to better characterize the specific DA neuronal expression profile associated with the late-stage Parkinson's condition. The default "Map" mode analysis resulted in 10 significantly over/under-expressed segments, mapping on 8 different chromosomes for SN whole tissue and in 4 segments mapping on 4 different chromosomes for DA neurons. In conclusion, TRAM software allowed us to confirm the deregulation of some genomic regions and loci involved in key molecular pathways related to neurodegeneration, as well as to provide new insights about genes and non-coding RNA transcripts not yet associated with the disease.

## Introduction

Parkinson's disease (PD) is a common neurodegenerative disorders, the second after Alzheimer's disease (AD), with an estimated incidence of 1–2% in individuals over 60 years of age [1]. It has been widely demonstrated that the degeneration of the dopamine (DA)-synthesizing cells of the substantia nigra (SN) pars compacta cause the common motor and non-motor symptoms of PD [2]. Generally, the onset of symptoms is correlated with the loss of about 50–70% of DA neurons [3] and another pathological hallmark of PD is the presence of intraneuronal cytoplasmic inclusions (Lewy bodies) [1]. The development of PD usually leads to death in 10 years after diagnosis [4]. To date, even if novel therapeutic approaches are being investigated in order to slow or halt neuronal degeneration [5], the most efficient treatment of PD still remains the use of levodopa, to relieve PD motor symptoms by replacing the deficient neurotransmitter DA. Although the pathology of the disease is very complex and its etiology remains unknown, research has highlighted the pathological role of different factors, in addition to genetic predispositions.

Several loci and genes have been identified in Mendelian forms of PD [3], furthermore the application of genome-wide screening revealed a significant number of genes that might contribute to disease risk [6]. Increasing evidence suggests that also epigenetic mechanisms, such as DNA methylation, histone modifications, and small RNA-mediated mechanisms, could regulate the expression of PD-related genes [7, 8].

Gene expression analysis could help to relate a gene or a cluster of genes to a particular biological mechanism, normal or pathological. Technologies to examine whole-genome gene expression, have rapidly advanced since the first application of microarray technology in 1996 [9], including, nowadays, exon microarray analysis, and transcriptome RNA sequencing [10, 11]. DNA microarrays, in particular, is the most frequently used technique, and several gene expression studies have already been conducted on post-mortem brain tissues of PD patients, mainly from SN [12–14], but also from DA neurons isolated with laser capture microdissection (LMD) [15–17].

Since most of the results showed low concordance among involved genes and pathways, meta-analysis approaches have been conducted in order to find greater data convergence, and have suggested new insight into the pathways potentially altered during PD pathogenesis [18, 19].

In the present study, we attempt to contribute to a better definition of expression differences between PD and healthy controls using TRAM (Transcriptome Mapper) software, which is able to analyse a large amount of publicly available microarray data from independent studies. The software can integrate original methods for parsing, normalizing, mapping, and statistically analyzing expression data conducted on different platforms [20]. In addition, it has the ability to easily generate maps showing differential expression between two sample groups, relative to two different biological conditions, pointing out chromosomal segments and statistically significant single gene loci [20].

Our meta-analysis was conducted on PD patients and controls microarray data obtained from the SN brain region, analysing both post-mortem whole tissue and isolated LMD DA neurons expression data, with the aim to specify the neuronal transcription signals.

## Materials and Methods

### Database search and selection

Gene Expression Omnibus (GEO) [21] functional genomics repository was searched for: "Parkinson disease" AND "Homo sapiens" [organism].

ArrayExpress database [22] of functional genomics experiments was searched at: http://www.ebi.ac.uk/arrayexpress/ for the term "Parkinson disease" and filtered for "Homo Sapiens" [by organism], "rna assay" and "array assay" [by experiment type] and “all array” [by array].

Filters for inclusion and exclusion of datasets in the analysis were applied as described in TRAM guidelines [20]. In particular, all the selected experiments were carried out on specific brain structure and clinical conditions (substantia nigra pars compacta from post-mortem brains or laser captured human dopaminergic neuron from individuals with PD and matched controls), based on availability of raw or pre-processed data.

Data from exon array or other probes were excluded, as a too high number of data rows could hinder the program execution. Other exclusion criteria were: the absence of identifiers corresponding to those found in the GEO sample records (GSM) or Array express sample records; platforms without standard format (for example with an atypical number of genes, i.e. <5.000 or >60.000); data whose expression values were not clearly identifiable as linear or logarithmic.

Searches were executed up to March 2015.

### Literature Search

A systematic biomedical literature search was performed up to March 2015 in order to identify articles related to global gene expression profile experiments in PD patients. First, a general search using the common terms "Parkinson disease" and "microarray analysis" was carried out.

Then, the MeSH terms "Parkinson disease", "microarray analysis" (or "gene expression profiling" or "oligonucleotide array sequence analysis"), "substantia nigra" and "human" were also used for a more advanced PubMed search.

All articles were cross-checked with database search results to find any additional available microarray data.

### TRAM analysis

TRAM (Transcriptome Mapper) software is freely available at http://apollo11.isto.unibo.it/software.

We used a version of TRAM including updated UniGene and Entrez Genes databases (TRAM 1.2, April 2014), in comparison with the original 2011 version [20].

For each series, we have downloaded the samples selected for the study in.txt format and subsequently they were divided in pools, based on the extraction method, in order to conduct the following analysis: TRAM SN ONLY, comparing the transcriptome map of whole substantia nigra of PD patient (pool A) and healthy control (pool B); TRAM DA ONLY, comparing the transcriptome map of laser microdissected DA neurons of PD patient (pool C) and healthy control (pool D).

In addition, the platforms not included in TRAM 1.2 version were manually extracted and imported. This step is required to associate the correct gene symbol to the probe identifiers in each experimental data set.

Finally, all samples grouped into folders for each pool, were imported in TRAM and automatically normalized by intra- and inter-sample normalization with default parameters [20]. Briefly, during expression data import all the gene or probe identifiers were converted to gene symbols via UniGene and then gene expression values were assigned to individual loci. According to the TRAM Guide available within the software, the intra-sample normalization was conducted with “Mean” default parameters, expressing each value as the percentage of the corresponding sample mean value. Likewise, the inter-sample normalization was conducted with “Scaled-Q” default parameters, a variant of quantile normalization useful to normalize data from platforms with highly different numbers of investigated genes [20].

For each locus, in each biological condition, TRAM calculated the expression value as the mean of all available values for that locus. The statistical significance was calculated taking into account all genes in the genome (genome median), in order to determine percentile thresholds to select over/under-expressed genes.

For both studies (TRAM SN ONLY and TRAM DA ONLY) we run the standard analysis "Map" mode, using default and single gene level parameters [20, 23].

In "Map" mode with default parameters, TRAM searched for over/under-expressed segments which have a window size of 500,000 bp and a shift of 250,000 bp, defining a segment as over/under-expressed in a significative manner if the expression value was different between the two conditions and contained at least 3 over/under-expressed genes (genes at the top/bottom 2.5% of values). In "Map" mode with single gene level parameters, the window size was set to12,500 bp with a shift of 6,250 bp, which corresponds to about a quarter of the mean lenght of a gene. In this way the significant over/under-expression of a segment corresponds in most cases to that of a single gene.

The software used in our study should assess the possible risk of bias, as it is intrinsically resistant to the systematic differences between batches (groups) of samples, as previously described [20].

### Other analysis

EBI Expression Atlas (http://www.ebi.ac.uk/gxa/home) [24], UniGene (http://www.ncbi.nlm.nih.gov/unigene) [25], NCBI Entrez Gene (www.ncbi.nlm.nih.gov/entrez/query.fcgi?db=gene) [26] and Gene Ontology [27], were used to obtain gene-specific information and to functionally characterize the large set of genes derived from the TRAM analysis.

## Results

### Database and literature search

GEO and Array Express wide search resulted in 71 series of expression data. We then filtered our analysis with criteria "substantia nigra" as specific area of interest and inclusion-exclusion restrictions (see Material and Methods section), achieving a total of 11 series. An additional data set was retrieved by the advanced PubMed search and kindly provided after correspondence with the author [16], see Table 1.

**Table 1:** Description of the main features of the samples used in TRAM analysis.

GEO Series	GEO Platform ID	Samples	CTR/PD	RNA Source	Age at death (range)	Pool	PMID
GSE25931	GPL13497	2/4	CTR	SN	N/A	B	22163301 [28]
GSE26927	GPL6255	8/20	CTR	SN	64.5 (54–104)	B	22864814 [29]
GSE26927	GPL6255	12/20	PD	SN	81.5 (76–87)	A	22864814 [29]
GSE20333	GPL201	6/12	CTR	SN	79 (68–88)	B	15455214 [12]
GSE20333	GPL201	6/12	PD	SN	77 (70–87)	A	15455214 [12]
GSE8397	GPL96	15/39	CTR	SN	69.9 (46–81)	B	16344956 [14]
GSE8397	GPL96	24/39	PD	SN	80.3 (68–89)	A	16344956 [14]
GSE8397	GPL97	15/39	CTR	SN	69.9 (46–81)	B	16344956 [14]
GSE8397	GPL97	24/39	PD	SN	80.3 (68–89)	A	16344956 [14]
GSE7621	GPL570	9/25	CTR	SN	N/A	B	17571925 [13]
GSE7621	GPL570	16/25	PD	SN	N/A	A	17571925 [13]
GSE20292	GPL96	18/29	CTR	SN	66.8 (41–94)	B	15965975 [30]
GSE20292	GPL96	11/29	PD	SN	75.4 (67–84)	A	15965975 [30]
GSE20164	GPL96	5/11	CTR	SN	80.6 (72–90)	B	20926834 [19]
GSE20164	GPL96	6/11	PD	SN	81,5 (74–87)	A	20926834 [19]
GSE20163	GPL96	9/17	CTR	SN	69.4 (52–84)	B	20926834 [19]
GSE20163	GPL96	8/17	PD	SN	78.5 (70–84)	A	20926834 [19]
GSE20159	GPL6947	17/33	CTR	SN	74.7 (40–95)	B	20926834 [19]
GSE20159	GPL6947	16/33	PD	SN	82.6 (56–103)	A	20926834 [19]
GSE24378	GPL1352	8/17	CTR	DA	71.7 (62–89)	D	20926834 [19]
GSE24378	GPL1352	9/17	PD	DA	76 (66–94)	C	20926834 [19]
GSE20141	GPL570	8/18	CTR	DA	N/A	D	20926834 [19]
GSE20141	GPL570	10/18	PD	DA	N/A	C	20926834 [19]
*	GPL96	9/19	CTR	DA	75.2 (68–89)	D	19052140 [16]
*	GPL96	10/19	PD	DA	78.4 (71–84)	C	19052140 [16]

Samples selected for the meta-analysis of gene expression profiles of PD patients vs. healthy controls. **GEO Series and Platform ID**: IDs numbers as reported in GEO database; **Samples**: number of samples selected for the meta-analysis; **CTR/PD**: control/PD patient sample; **RNA source**: brain tissue and method of extraction (SN: substantia nigra whole tissue; DA: dopaminergic neurons from laser capture microdissection); **Age at death**: mean value of all the indicated patient/control age of death (the age range was also reported); **N/A**: not available; **Pool**: all samples were divided into 4 pools, based on the extraction method in order to perform the analysis TRAM DA ONLY and TRAM SN ONLY (see Results section). **PMID:** PubMed identifier number of the reference reported in GEO database.

* The serie is not deposited in GEO database [see Materials and Methods section]. More details about samples are listed in S1 Table.

**Fig 1:**
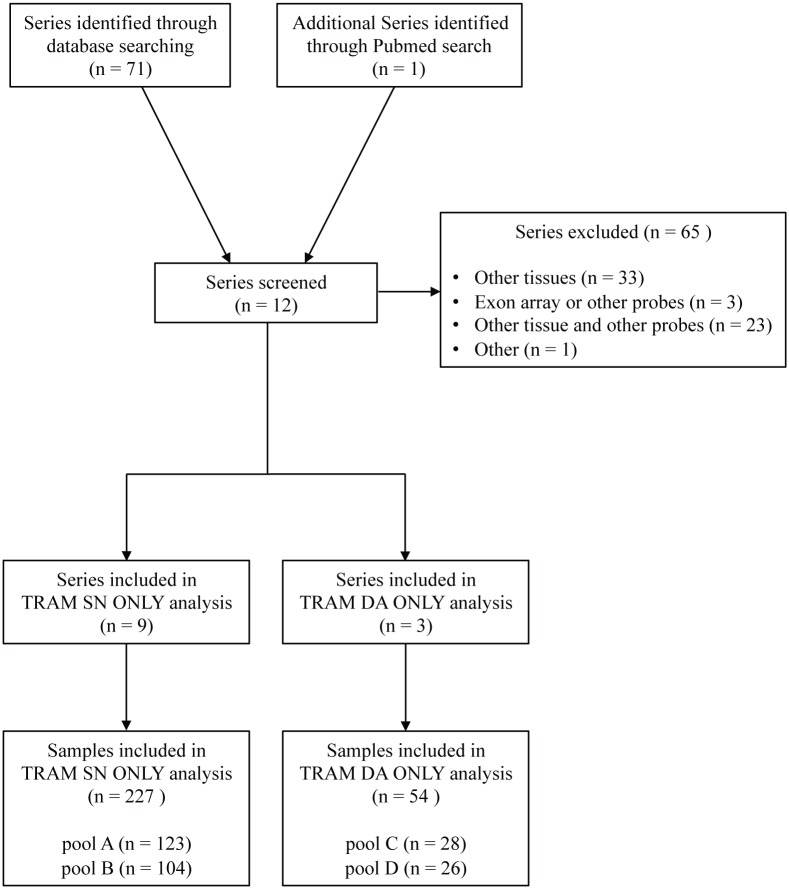
Flow diagram of data searching and selection strategy for TRAM meta-analysis.

According to RNA source, 9 series were included in the TRAM SN ONLY analysis and 3 series were included in the TRAM DA ONLY analysis (Fig 1 and Table 1).

The total number of retrieved samples was 151 patients with symptomatic Parkinson's and subclinical disease (iLBD: incidental Lewy Bodies Disease [31]), and 130 samples as age-matched controls. According to the different tissue collection (snap-frozen or laser capture microdissection) they were further subdivided in: pool A (PD patients SN ONLY); pool B (controls SN ONLY); pool C (PD patients DA ONLY); pool D (controls DA ONLY).

A complete description of sample identifiers and main sample features are listed in Table 1 and S1 Table. The selection strategy for eligible data is summarized in Fig 1.

### Snap-frozen SN tissue PD patients vs. controls

We first analyzed differential expression of pool A (123 PD substantia nigra samples) versus pool B (104 control substantia nigra samples), derived from 9 series of expression data.

A total of 3,166,787 data points (gene expression value) from the pool A and 2,643,992 data points from the pool B, relative to 36,446 distinct loci for which an A/B ratio value was determinable, were included in the analysis (S2 Table).

"Map" mode analysis of pool A vs. pool B data resulted in 10 significantly over/under-expressed segments, mapping on 8 different chromosomes (Table 2, SN ONLY).

**Table 2:** List of the over/under-expressed segments and genes generated by TRAM "Map" mode analysis.

Chr	Location	Segment Start	Segment End	Expression Ratio	p value	q value	Genes in the segment
**SN ONLY**							
chr1	1p36*	22,250,001	22,750,000	1.21	0.0016	0.0022	*Hs*.*538178- ZBTB40+ Hs*.*670193+ EPHA8+* ***C1QA+ C1QC+ C1QB+*** *Hs*.*538176- Hs*.*563960+ EPHB2-*
chr7	7p15	23,250,001	23,750,000	1.16	0.0002	0.0009	***GPNMB+*** *MALSU1+ IGF2BP3+ Hs*.*29733- TRA2A+* ***Hs*.*644466+*** *Hs*.*608901+* ***Hs*.*743502+*** *CCDC126+ Hs*.*737536+ Hs*.*128757+* ***FAM221A+*** *STK31-*
chr3	3q13.31	114,250,001	114,750,000	1.13	0.0029	0.0033	***TIGIT- Hs*.*592414+*** *ZBTB20+* ***Hs*.*744879+*** *Hs*.*193784+ Hs*.*614383- Hs*.*732516+* ***Hs*.*202577+*** *ZBTB20-AS1- Hs*.*659543+ Hs*.*655764- Hs*.*663956+*
chr1	1p22.2	89,000,001	89,500,000	1.12	0.0046	0.0046	***GBP3+ GBP1+*** *Hs*.*205458+ Hs*.*170957-* ***Hs*.*732899+*** *GBP2+ GBP7- GBP4+ Hs*.*562189+ GBP5+ GBP6- Hs*.*563877+ Hs*.*537991+ Hs*.*432947+*
chr16	16q13	56,250,001	56,750,000	1.12	<0.0005	<0.0002	*GNAO1+* ***Hs*.*666766+*** *AMFR- NUDT21+ OGFOD1- BBS2+ MT4- MT3+* ***MT2A+*** *Hs*.*569566- MT1L+ MT1E+* ***MT1M+*** *MT1A+ MT1B-* ***MT1F+ MT1G+ MT1H+ MT1X+*** *Hs*.*724197+ NUP93-*
chr13	13q12.12 *	24,750,001	25,250,000	1.11	0.0016	0.0022	*Hs*.*572245- RNF17- CENPJ+ Hs*.*737044+* ***Hs*.*731897+ PABPC3+ AMER2+*** *Hs*.*585620+ Hs*.*577996- MTMR6-*
chr17	17p12	9,500,001	10,000,000	0.72	0.0012	0.0016	*STX8-* ***WDR16- USP43-*** *Hs*.*594758+ DHRS7C- GLP2R- Hs*.*562746-* ***RCVRN- GAS7+***
chr11	11p15.4*	4,500,001	5,000,000	0.71	0.0017	0.0017	*Hs*.*425805- OR52M1- C11orf40-* ***OR52I2-*** *OR52I1- TRIM68+ OR51D1- OR51E1- OR51E2- OR51F1- OR52R1- OR51F2- OR51S1- OR51T1+* ***OR51A7-*** *OR51G2-* ***OR51G1-*** *OR51A4+* ***OR51A2-*** *MMP26- OR51L1-*
chr7	7q11.21	65,750,001	66,250,000	0.68	0.0005	0.0010	***LOC441242-*** *VKORC1L1- Hs*.*661342+ GUSB-* ***ASL-*** *CRCP+* ***TPST1-***
chr5	5q13.3	76,000,001	76,500,000	0.34	<0.0005	<0.0002	***SV2C- Hs*.*646953- IQGAP2-***
**DA ONLY**							
chr2	2q31-q32	172,750,001	173,250,000	2.03	0.0003	0.0012	*RAPGEF4- ZAK-* ***Hs*.*674047+ MLK7AS1+*** *Hs*.*713091-* ***Hs*.*663335+***
chr11	11p15.5	1,000,001	1,500,000	1.79	0.0016	0.0016	*AP2A2-* ***MUC6+ Hs*.*703727+*** *Hs*.*436626+ MUC2+* ***MUC5AC****+ MUC5B- TOLLIP- BRSK2- MOB2-*
chrX	Xp11.23	48,000,001	48,500,000	1.76	0.0012	0.0016	*ZNF182+ ZNF630+ SSX5+* ***SSX1****+ SSX3+* ***SSX4+ SSX4B+*** *SLC38A5+ FTSJ1-*
chr15	15q26.1*	92,750,001	93,250,000	0.74	0.0002	0.0002	***LOC100507217****-* ***Hs*.*741028****- CHD2+ Hs*.*709650-* ***RGMA-***

"Map" mode analysis results of PD patients vs. controls SN ONLY (pool A vs. pool B) and PD patients vs. controls DA ONLY (pool C vs. pool D). The over/under-expressed segments were retrieved by genome median analysis, performed using default parameters (see Materials and Methods section). Segments are sorted by decreasing A/B or C/D ratio. TRAM displays UniGene EST clusters (with the prefix “Hs.” in the case of Homo sapiens) only if they have an expression value. **Chr**: chromosome; **Location**: segment cytoband derived from that of the first mapped gene within the segment; **Segment Start/End**: chromosomal coordinates for each segment; **Genes in the segment**: bold and +: over-expressed gene; bold and -: under-expressed gene; '+' or '-': gene expression value higher or lower than the median value, respectively.

*Cytoband was derived from the UCSC Genome Browser (http://genome-euro.ucsc.edu/cgi-bin/hgGateway)

The higher expression ratio between PD patients and controls substantia nigra whole tissue was observed in chromosome 1 [coordinates 22,250,001–22,750,000], with three known genes of the human complement subcomponent C1q showing values within the higher 2.5th percentile: *C1QA* and *C1QB* and *C1QC*. Then TRAM analysis retrieved a segment on chromosome 7 [spanned from coordinates 23,250,001–23,750,000], containing two sequences referred to EST clusters, Hs.644466 and Hs.743502 and the *FAM221A* gene, encodes for a protein with unknown function. Analysis conducted on UniGene and EBI database indicate that *FAM221A* is highly conserved from human to zebrafish, with a specific expression in brain tissue. In the same segment, it is over-expressed also the known gene *GPNMB*, encoding for a type I transmembrane glycoprotein.

The third over-expressed segment was on chromosome 3 [coordinates 114,250,001–114,750,000], with the known gene *TIGIT*, encoding for a member of the PVR (poliovirus receptor) family of immunoglobin proteins, and the three EST cluster (Hs.592414, Hs.744879 and Hs.202577) which show an increase of expression in PD patients compared to controls.

Other three over-expressed segments were: on chromosome 1 [coordinates 89,000,001–89,500,000], including as statistically significant two known genes of the GBP family of guanylate binding protein, *GBP3* and *GBP1*, and one EST cluster: Hs.732899; on chromosome 16 [coordinates 56,250,001–56,750,000], with several metallothionein superfamily genes marked as significative; on chromosome 13 [coordinates 24,750,001–25,250,000], with two known genes, *PABPC3* and *AMER2* encoding respectively for a poly(A) binding protein and for an APC membrane recruitment protein 2 and the EST cluster Hs.731897, exhibiting an altered expression. The first under-expressed segment, with the lowest expression value in PD patients, was located on chromosome 5 [coordinates 76,000,001–76,500,000] (Table 2), with two known genes that are characterized by a statistically significant under-expression in substantia nigra of PD patients: *SV2C* and *IQGAP2*, encoding respectively for synaptic vesicle glycoprotein 2C and a member of the IQGAP (IQ motif containing GTPase activating protein) family. The altered expression was also observed for the EST cluster Hs.646953. Then, there was the genomic segment on chromosome 7 [coordinates 65,750,001–66,250,000], containing one ncRNA (*LOC441242*) and two known loci, *ASL* and *TPST1*, encoding respectively for a member of the lyase 1 family and for the tyrosylprotein sulfotransferase 1. The third under-expressed segment spans the cluster of olfactory receptor genes located on chromosome 11 in the 11p15.4 region [coordinates 4,500,001–5,000,000], with some isoforms marked as statistically significant: *OR52I2*, *OR51A7*, *OR51G1* and *OR51A2*. Finally, the last under-expressed segment, with the same expression ratio of the previous one, was on chromosome 17 [coordinates 9,500,001–10,000,000]. The statistical significance was obtained for three known genes: *WDR16*, encodes for a WD repeat-containing proteins, *USP43* an ubiquitin specific peptidase 43 and *RCVRN* encoding for a member of the recoverin family of neuronal calcium sensors. The segment also includes an over-expressed gene *GAS7*, which encodes for a growth arrest-specific 7 protein.

At single gene level (TRAM segment window of 12,500 bp), the known gene *DEFA3*, defensin, alpha 3 neutrophil-specific protein (chr8), has the highest expression value (Table 3 and S3 Table), followed by two known gene, *AZGP1* (chr7) and *PCDH20* (chr13), encoding respectively for alpha-2-glycoprotein 1, zinc-binding, and for a member of the protocadherin gene family, a subfamily of the cadherin superfamily.

**Table 3:** Top twenty list of genes significantly over- or under-expressed in SN ONLY.

Genes	Chr	Expression value A	Expression value B	A/B	p value	q value	Data points A	Data points B	SD% A	SD% B	GO term Process
**OVER-EXPRESSED GENES**											
*DEFA3*	chr8	103.37	20.96	4.93	0.0250	0.0260	28	27	388.81	87.01	innate immune response (GO:0045087)
*AZGP1*	chr7	126.99	57.63	2.20	0.0250	0.0260	166	143	109.93	108.28	immune response (GO:0006955)
*PCDH20*	chr13	133.73	62.01	2.16	0.0250	0.0260	68	51	99.42	95.18	cell adhesion (GO:0007155)
*CTSG*	chr14	42.59	19.83	2.15	0.0250	0.0260	100	88	480.67	51.89	angiotensin maturation (GO:0002003)
*NPTX2*	chr7	261.40	122.44	2.13	0.0250	0.0260	94	82	87.34	102.19	synaptic transmission (GO:0007268)
*Hs*.*291993*	chr13	80.19	37.69	2.13	0.0250	0.0260	56	41	109.12	45.91	*uncharacterized*
*SLC38A2*	chr12	1,015.73	477.45	2.13	0.0250	0.0260	206	169	114.33	118.89	amino acid transport (GO:0006865)
*BOK*	chr2	296.41	141.19	2.10	0.0250	0.0260	140	112	153.60	133.83	apoptotic process (GO:0006915)
*USP54*	chr10	675.24	327.69	2.06	0.0250	0.0260	68	51	81.51	86.91	protein deubiquitination (GO:0016579)
*DEFA1*	chr8	92.03	45.26	2.03	0.0250	0.0260	148	137	378.79	368.04	innate immune response (GO:0045087)
*USP31*	chr16	316.63	159.36	1.99	0.0250	0.0260	164	110	91.95	83.16	protein deubiquitination (GO:0016579)
*LINC00844*	chr10	2,425.15	1,223.60	1.98	0.0250	0.0260	56	41	85.85	97.98	*ncRNA*
*DANCR*	chr4	305.40	160.14	1.91	0.0250	0.0260	40	26	39.04	30.96	*uncharacterized*
*LRP2*	chr2	130.19	68.32	1.91	0.0250	0.0260	139	113	108.13	84.02	Wnt signaling pathway (GO:0060070)
*DOCK5*	chr8	171.88	91.12	1.89	0.0250	0.0260	310	231	147.56	147.90	positive regulation of GTPase activity (GO:0043547)
*PDK4*	chr7	347.20	184.22	1.88	0.0250	0.0260	155	122	125.33	129.28	cellular metabolic process (GO:0044237)
*SLC5A11*	chr16	352.10	188.32	1.87	0.0250	0.0260	68	51	58.45	59.75	apoptotic process (GO:0006915)
*MAFK*	chr7	117.65	63.73	1.85	0.0250	0.0260	139	113	170.50	159.09	regulation of transcription(GO:0006357)
*PTGS2*	chr1	2,685.97	1,479.78	1.82	0.0250	0.0260	163	133	204.02	250.58	NAD metabolic process (GO:0019674)
*CDK2AP2*	chr11	106.81	59.57	1.79	0.0250	0.0260	99	89	71.57	56.51	N/A
**UNDER-EXPRESSED GENES**											
*NHLRC1*	chr6	12.33	84.44	0.15	0.0250	0.0252	28	27	21.35	397.11	protein polyubiquitination (GO:0000209)
*C12orf50*	chr12	14.49	102.57	0.14	0.0250	0.0252	84	70	70.81	685.40	nucleic acid binding (GO:0003676)
*SIRPD*	chr20	25.92	185.50	0.14	0.0250	0.0252	68	53	35.55	623.70	N/A
*WNT9B*	chr17	15.56	112.31	0.14	0.0250	0.0252	44	36	64.63	495.38	Wnt signaling pathway (GO:0060070)
*REG4*	chr1	13.16	101.70	0.13	0.0250	0.0252	84	62	40.77	669.15	carbohydrate binding (GO:0030246)
*OR51G1*	chr11	13.23	104.38	0.13	0.0250	0.0252	28	27	28.50	312.26	signal trasduction (GO:0007165)
*IQCF6*	chr3	12.95	105.92	0.12	0.0250	0.0252	40	26	35.21	441.65	N/A
*CFAP54*	chr12	15.62	128.26	0.12	0.0250	0.0252	44	40	45.56	532.52	integral component of membrane
*KDF1*	chr1	13.87	121.06	0.11	0.0250	0.0252	68	53	55.21	619.20	developmental growth (GO:0048589)
*KIAA0087*	chr7	10.14	89.23	0.11	0.0250	0.0252	65	62	38.06	679.79	*ncRNA*
*CLC*	chr19	16.14	153.92	0.10	0.0250	0.0252	100	88	87.05	825.34	apoptotic process (GO:0006915)
*CDY2A*	chrY	18.56	177.17	0.10	0.0250	0.0252	28	27	46.12	460.82	histone acetylation (GO:0016573)
*MAGEA2B*	chrX	12.85	134.08	0.10	0.0250	0.0252	28	29	37.22	459.70	negative regulation of protein acetylation (GO:1901984)
*COLCA2*	chr11	48.63	537.78	0.09	0.0250	0.0252	40	26	32.55	456.61	N/A
*FOXB2*	chr9	14.85	169.39	0.09	0.0250	0.0252	28	27	53.82	453.21	cell differentiation (GO:0030154)
*ANGPTL5*	chr11	14.78	231.16	0.06	0.0250	0.0252	28	27	32.65	463.92	N/A
*ARSH*	chrX	16.93	267.32	0.06	0.0250	0.0252	28	27	62.76	473.06	metabolic process (GO:0008152)
*PSMG3-AS1*	chr7	32.90	625.84	0.05	0.0250	0.0252	16	13	34.45	334.15	*ncRNA*
*TEX22*	chr14	19.35	428.53	0.05	0.0250	0.0252	16	11	32.01	313.27	N/A
*OR8H3*	chr11	32.63	813.20	0.04	0.0250	0.0252	28	27	74.87	495.50	signal trasduction (GO:0007165)

The twenty most over- and under-expressed genes resulted in SN ONLY (pool A vs. pool B) "Map" mode analysis with a segment window of 12,500 bp, considering genome median analysis (see full results in Supplementary Information section). **Data points**: number of spots related to an expression value for the locus. **SD**: standard deviation of the expression value indicated as percentage of the mean. **GO term Process**: description and accession number of the main biological process associated to the gene according to Gene Ontology Consortium. **N/A**: not available in the Gene Ontology database.

Between the under-expressed genes in PD patients tissue, a fold increase lower than 20 is observed for three genes, in particular *OR8H3*, another gene of the olfactory receptor family, *TEX22* named testis expressed 22, a protein coding gene with unknown function to date, and the ncRNA *PSMG3 –AS1*. Single gene level analysis of SN whole tissue data generated a total of 11,775 significative loci (see S3 Table for the complete list of over/under-expressed genes), corresponding to 1,217 single transcripts with altered expression.

### Laser microdissected DA neurons PD patients vs. controls

The TRAM analysis of the 3 series of laser microdissected tissue expression data, pool C (28 PD neurons samples) vs. pool D (26 control neurons samples), processed a total of 1,098,430 data points from the pool C and 1,035,561 data points from the pool D, relative to 25,795 distinct loci for which an C/D ratio value was determinable (S4 Table).

"Map" mode analysis of pool C vs. pool D data resulted in 4 segments with statistical significance, mapping on 4 different chromosomes (Table 2, DA ONLY). Three of the four segments show an over-expression and only one includes genes that are under-expressed in PD patients DA neurons.

The higher expression ratio between PD patient and control neurons was observed in chromosome 2 [coordinates 172,750,001–173,250,000], with one ncRNA *MLK7-AS1* and two EST clusters marked as statistically significant. Then the second most over-expressed segment was on chromosome 11 [coordinates 1,000,001–1,500,000] and spanned the cluster of mucin genes in the 11p15.5 region, with an over-expression observed only for *MUC6* and *MUC5AC* genes. The last segment is on chromosome X [coordinates 48,000,001–48,500,000], with three known genes belonging to the family of highly homologous synovial sarcoma X (SSX) breakpoint proteins showed expression values within the higher 2.5th percentile: *SSX1*, *SSX4* and *SSX4B*.

Finally, the only segment resulted under-expressed in PD patients DA neurons was on chromosome 15 [coordinates 92,750,001–93,250,000] (Table 2), and included one known gene *RGMA* encoding for a glycosylphosphatidylinositol-anchored glycoprotein, the *LOC100507217* locus encoding for a ncRNA and the sequence named Hs.741028, which is to date uncharacterized.

At single gene level (TRAM segment window of 12,500 bp), the long non-coding *LINC00520* mapping on chromosome 14 has the highest expression value (Table 4 and S5 Table), followed by two EST clusters (Hs.512440, Hs.554217) mapping respectively on chromosome 8 and chromosome 20. Fold increase higher than 4 is observed also for *MUC4* and *MUC6* genes, located respectively on chromosome 3 and 11, belonging to the mucin family, and for *LYG2* on chromosome 2, encoding for a protein with lysozyme activity. Then also the ncRNA, *MLK7-AS1* and the 3 EST cluster: Hs.291993, Hs.618995 and Hs.630709, show a similar fold increase.

**Table 4:** Top twenty list of genes significantly over- or under-expressed in DA ONLY.

Genes	Chr	Expression value C	Expression value D	C/D	p value	q value	Data points C	Data points D	SD% C	SD% D	GO terms Process
**OVER-EXPRESSED GENES**											
*LINC00520*	chr14	208.92	20.94	9.97	0.0250	0.0310	10	8	228.81	77.82	*ncRNA*
*Hs*.*512440*	chr8	96.55	14.25	6.77	0.0250	0.0310	28	25	273.12	99.36	*ncRNA (LOC101929450)*
*Hs*.*554217*	chr20	160.33	30.27	5.30	0.0250	0.0310	18	17	165.83	66.03	*uncharacterized*
*MUC4*	chr3	153.37	32.08	4.78	0.0250	0.0310	130	120	491.76	166.80	cell-matrix adhesion (GO:0007160)
*LYG2*	chr2	84.86	18.05	4.70	0.0250	0.0310	18	17	251.16	125.75	peptidoglycan catabolic process (GO:0009253)
*MUC6*	chr11	236.13	54.42	4.34	0.0250	0.0310	46	43	319.68	71.83	maintenance of gastrointestinal epithelium (GO:0030277)
*Hs*.*291993*	chr13	219.13	50.55	4.34	0.0250	0.0310	18	17	232.95	82.03	*uncharacterized*
*MLK7-AS1*	chr2	24.02	5.86	4.10	0.0250	0.0310	18	17	133.39	135.70	*ncRNA*
*Hs*.*618995*	chr12	91.51	22.70	4.03	0.0250	0.0310	18	17	176.37	75.38	*uncharacterized*
*Hs*.*630709*	chr2	212.81	53.10	4.01	0.0250	0.0310	18	17	130.27	180.07	*uncharacterized*
*DEFB108B*	chr11	88.26	22.12	3.99	0.0250	0.0310	18	17	139.62	108.56	innate immune response (GO:0045087)
*Hs*.*411959*	chr18	64.76	16.45	3.94	0.0250	0.0310	18	17	145.20	100.31	*uncharacterized*
*FAM87A*	chr8	139.26	35.56	3.92	0.0250	0.0310	28	25	267.62	110.05	*ncRNA*
*TRIM54*	chr2	42.51	10.94	3.89	0.0250	0.0310	18	17	116.14	126.09	microtubule-based process (GO:0007017)
*LINC00202-1*	chr10	75.01	19.53	3.84	0.0250	0.0310	18	17	97.94	88.61	*ncRNA*
*SHISA7*	chr19	124.07	33.52	3.70	0.0250	0.0310	18	17	125.02	66.82	short-term neuronal synaptic plasticity (GO:0048172)
*GAS2L3*	chr12	80.59	22.00	3.66	0.0250	0.0310	18	17	135.74	69.88	cytoskeleton organization (GO:0007010)
*IRX2*	chr5	172.81	47.60	3.63	0.0250	0.0310	44	43	287.53	146.46	regulation of transcription (GO:0006357)
*Hs*.*216363*	chr1	49.45	13.64	3.63	0.0250	0.0310	18	17	162.98	103.74	*ncRNA (LOC101927342)*
*Hs*.*661268*	N/A	142.91	41.04	3.48	0.0250	0.0310	18	17	150.42	93.33	*uncharacterized*
**OVER-EXPRESSED GENES**											
*RNF166*	chr16	41.14	109.18	0.38	0.0250	0.0252	18	17	63.31	289.31	protein polyubiquitination (GO:0000209)
*IP6K3*	chr6	44.87	121.82	0.37	0.0250	0.0252	18	17	79.95	117.30	protein phosphorylation (GO:0006468)
*CGNL1*	chr15	194.95	530.97	0.37	0.0250	0.0252	18	17	107.64	167.00	metabolic process (GO:0008152)
*BORCS5*	chr12	14.68	40.14	0.37	0.0250	0.0252	18	17	123.78	155.22	N/A
*MYOM1*	chr18	285.90	785.14	0.36	0.0250	0.0252	28	26	160.76	161.70	mitophagy (GO:0000422)
*PCP4L1*	chr1	43.42	119.96	0.36	0.0250	0.0252	18	17	72.83	299.54	N/A
*Hs*.*598973*	N/A	22.43	64.60	0.35	0.0250	0.0252	18	17	64.50	305.81	*uncharacterized*
*AGTR1*	chr3	49.45	146.86	0.34	0.0250	0.0252	56	52	89.01	195.05	signal transduction (GO:0007165)
*ANKRD33*	chr12	27.67	82.28	0.34	0.0250	0.0252	18	17	60.61	307.02	negative regulation of transcription (GO:0000122)
*SPSB4*	chr3	33.22	100.13	0.33	0.0250	0.0252	18	17	121.09	319.24	signal trasduction (GO:0007165)
*ZAN*	chr7	18.89	57.83	0.33	0.0250	0.0252	58	49	97.97	401.99	binding of sperm to zona pellucida (GO:0007339)
*LINC00641*	chr14	36.25	111.22	0.33	0.0250	0.0252	18	17	119.83	268.49	*ncRNA*
*C10orf35*	chr10	77.74	240.21	0.32	0.0250	0.0252	18	17	73.18	154.94	N/A
*BEX5*	chrX	125.56	398.29	0.32	0.0250	0.0252	18	17	122.61	177.33	N/A
*GLDN*	chr15	78.97	260.72	0.30	0.0250	0.0252	36	34	118.60	319.67	Nav channel clustering (GO:0045162)
*LOC100129603*	chr7	21.71	72.14	0.30	0.0250	0.0252	10	8	114.83	239.89	*ncRNA*
*LOC105377468*	chr4	20.80	71.08	0.29	0.0250	0.0252	18	17	88.16	268.02	*ncRNA*
*CARS*	chr11	161.53	722.23	0.22	0.0250	0.0252	110	103	274.50	485.11	cysteinyl-tRNA aminoacylation (GO:0006423)
*MIR622*	chr13	25.24	117.88	0.21	0.0250	0.0252	20	17	48.97	327.29	*ncRNA*
*ALDH1A1*	chr9	182.50	886.84	0.21	0.0250	0.0252	28	26	164.32	306.89	cellular aldehyde metabolic process (GO:0006081)

The twenty most over- and under-expressed genes resulted in DA ONLY (pool C vs. pool D) "Map" mode analysis with a segment window of 12,500 bp, considering genome median analysis (see full results in Supplementary Information section). **Data points**: number of spots related to an expression value for the locus. **SD**: standard deviation of the expression value indicated as percentage of the mean. **GO term Process**: description and accession number of the main biological process associated to the gene according to Gene Ontology Consortium. **N/A**: not available in the Gene Ontology database.

Among the most under-expressed genes in PD patients DA neurons there are several genes that show a fold decrease from 3 to almost 5, including the known genes *ALDH1A1* (chr9), encoding for a member of aldehyde dehydrogenase family, the cysteinyl-tRNA synthetase gene *CARS* (chr11), *GLDN* (chr15), encoding for gliomedin and *BEX5* (Brain Expressed, X-Linked 5).

Single gene level analysis of DA neurons data generated a total of 7,342 significative loci (see S5 Table for the complete list of over/under-expressed genes), corresponding to 759 single transcripts.

### Comparison with previously published data

We compared our results with the ones obtained in the individual works that were included in TRAM meta-analysis. We selected the main genes resulted as differentially expressed in the previously published microarray studies and verified their expression profile in our analysis (S6 Table).

In Table 5 genes from at least two independent single studies are listed, with the expression values obtained from our two differential maps. A general trend of over/under-expression consistent with data available in the literature is confirmed (see references indicated).

**Table 5:** Comparison with previously published data.

Genes	Chr	A/B (SN)	C/D (DA)	References	GO term Process
*AGTR1*	chr3	**0.46**	**0.34**	[13, 29, 30]	signal transduction (GO:0007165)
*ALDH1A1*	chr9	**0.41**	**0.21**	[12, 13, 29, 30]	cellular aldehyde metabolic process (GO:0006081)
*ANK1*	chr8	**0.43**	0.71	[13, 19, 29, 30]	cytoskeleton organization (GO:0007010)
*ATP5J*	chr21	0.93	**0.48**	[16, 19]	mitochondrial proton transport (GO:0042776)
*ATP5L*	chr11	0.99	**0.59**	[16, 19]	mitochondrial proton transport (GO:0042776)
*ATP6V1D*	chr14	0,89	**0.57**	[19, 30]	proton transport (GO:0015992)
*BEX1*	chrX	0.70	**0.41**	[14, 19, 29, 30]	up regulation of transcription factor (GO:0045944)
*CBLN1*	chr16	**0.52**	0.70	[13, 29, 30]	synaptic transmission (GO:0007268)
*COX6C*	chr8	1.02	**0.51**	[16, 19]	metabolic energy generation (GO:0006091)
*DNM1*	chr9	0.73	**0.60**	[16, 19]	endocytosis (GO:0006897)
*DYNC1I1*	chr7	0.68	**0.53**	[16, 19]	vesicle transport along microtubule (GO:0047496)
*FGF13*	chrX	**0.40**	0.69	[14, 19, 30]	MAPK cascade (GO:0000165)
*GABRB1*	chr4	**0.52**	0.72	[16, 29]	signal transduction (GO:0007165)
*HSPB1*	chr7	**1.63**	2.08	[14, 30]	intracellular signal transduction (GO:0035556)
*JMJD6*	chr17	**1.63**	1.22	[14, 30]	histone demethylation (GO:0016577)
*MKNK2*	chr19	**1.5**	1.15	[14, 30]	regulation of translation (GO:0006417)
*NDUFB2*	chr7	0.88	**0.44**	[16, 19]	complex I (NADH to ubiquinone) (GO:0006120)
*NPTX2*	chr7	**2.13**	1.42	[15, 29]	synaptic transmission (GO:0007268)
*RGS4*	chr1	**0.46**	**0.54**	[14, 30]	signal transduction (GO:0007165)
*SV2B*	chr15	**0.45**	0.82	[14, 16, 30]	neurotransmitter transport (GO:0006836)
*SYT1*	chr12	0.54	**0.58**	[14, 16, 19, 30]	synaptic transmission (GO:0007268)
*TF*	chr3	**1.33**	0.80	[14, 15, 30]	iron ion homeostasis (GO:0055072)
*TUBD1*	chr17	**1.29**	1.45	[15, 16]	microtubule-based process (GO:0007017)
*UQCRC2*	chr16	0.66	**0.55**	[12, 16, 19]	aerobic respiration (GO:0009060)
*ZBTB16*	chr11	**1.45**	1.63	[29, 30]	transcription, DNA-templated (GO:0006351)

The known genes confirmed in at least two independent single studies are reported (see references indicated). **Chr**: chromosome; **A/B (SN)** and **C/D (DA)**: expression ratio of value A/value B (SN ONLY) and value C/value D (DA ONLY) resulted from TRAM analysis (see respectively, S2 and S4 Tables). In bold: expression ratio values statistically significative in single gene level TRAM analysis, q value<0.05 (see respectively, S3 and S5 Tables); **GO term Process**: description and accession number of the main biological process associated to the gene according to Gene Ontology Consortium.

## Discussion

In this work, we proposed a transcriptome analysis of human substantia nigra, the most affected brain structure in Parkinson's disease. In particular, we have investigated the different expression profiles of PD patients brain compared to age-matched controls, considering data from snap frozen whole tissue as well as from isolated DA neurons, in distinct analyses. The aim was to better characterize the specific DA neuronal profile compared to the whole tissue section, including a large amount of cells other than DA neurons, as astrocytes, microglia and oligodendroglia cells. Usually, microarray analyses on dissected tissue revealed a set of deregulated genes, which is in agreement with the evidence that not only the DA neurons, but also other cells within the substantia nigra and adjacent brain regions, are involved in Parkinson's disease pathology [32].

The meta-analysis was conducted with TRAM software, a tool that can integrate data from different microarray experiments, performed on different platforms, through a method of intra- and inter-sample normalization (scaled quantile normalization), intrinsically not affected by the systematic differences between groups of samples in microarray experiments [20]. The problem of the batch effect and the statistical validity of TRAM has been previously discussed and confirmed in different recent studies [23, 33, 34].

We processed a relevant number of samples, derived from 9 series of expression data from post-mortem dissected tissue and 3 series of data from LMD DA neurons and the program allowed us to identify over/under-expressed critical genome regions, by comparing PD patients whole tissue vs. matched controls (pool A vs. pool B) or isolated DA cells vs. matched controls (pool C vs. pool D).

TRAM identifies critical genomic regions and genes with significant differential expressions between two biological conditions. In particular, it can be noted that when comparing PD isolated DA neurons vs. controls only a few significantly over- or under-expressed genomic regions are retrieved, indicating similarity between the transcriptome maps of the two conditions. It is well known that loss of neurons is considered a physiological condition typical of brain aging and post-mortem evidence suggests that the PD dopaminergic pathways are especially vulnerable to the effects of aging [35]. More recently also Elstner et al. have focused that PD expression profile of DA neurons, dramatically and specifically change when compared to younger control group instead of age-matched controls [17].

On the other hand, TRAM analysis highlights those particular regions that may discriminate the disease and that could therefore be essential for the identification of novel molecular pathways contributing to the pathogenesis of PD.

When comparing PD whole substantia nigra to that of age-matched controls, more genomic segments are retrieved by TRAM default analysis as a probable consequence of the SN tissue cell heterogeneity, and results obtained with both "Map" mode and single gene level analyses showed no overlapping data between SN and DA neuron expression profiles (Tables 2–4).

Moreover, a prevalent up-regulated activation of gliosis/inflammation specific genes is evident in the first analysis, likely due to late stages of PD patients.

Our results indicate that the most expressed segment in SN ONLY analysis is located on chromosome 1 (1p36), including *C1QA*, *C1QB* and *C1QC* genes, encoding for major constituents of the human complement subcomponent C1q (A chain, B chain and C chain, respectively). This seems to be consistent with the main presence of microglia cells in the sample, being the only cells that expressed C1q in SN and other brain areas [36]. It is known, in fact, that activation of the complement system promotes the removal of pathogens and tissue damage products from the brain and may be involved in neuronal cell death in neurodegenerative diseases. Besides, this observation is supported by recent results obtained in a mouse model of PD, showing that C1q is up-regulated in the nigrostriatal system [36].

Likewise, the second identified over-expressed segment in PD SN samples contain the known gene *GPNMB*, encoding a transmembrane protein, whose homologous has been shown to be implicated in the regulation of immune/inflammatory responses and expressed in microglia and macrophages in rat neural tissues [37]. Recent genome-wide meta-analysis studies have highlighted the *GPNMB* locus (7p15) as a new potential PD risk candidate gene [38]. Moreover, Tanaka and coll., provided evidence that GPNMB could have a potential protective role in neurodegenerative disorders, in particular in Amyotrophic Lateral Sclerosis (ALS) [39], suggesting that the locus should be further investigated also in PD models and human post-mortem tissue.

Other regions marked as over-expressed in affected SN tissue contain inflammation-related gene loci: *TIGIT* (3q13.31), recently investigated for its role in immune regulation, especially in cancer and other chronic disease [40]; *GBP3* and *GBP1* (1p22.2), already been reported as differentially expressed in post-mortem brain studies [41].

Significantly, it is well known that the chromosome 1 regions which resulted relevant in our meta-analysis, have been previously considered as a hotspot for Parkinson's disease genes [42, 43], confirming the efficiency of TRAM software. Another region of the genome identified as a locus for PD susceptibility and marked as the most under-expressed one in TRAM SN whole tissue analysis, is on chromosome 5 (5q13.3) [44]. In particular, the segment includes the known gene *SV2C*, encoding for the synaptic vesicle glycoprotein 2C, involved in neurotransmitter transport and densely expressed in dopaminergic neurons in substantia nigra [45]. A study by Nowack and coll. [46], has shown that modest changes in *SV2C* expression, in either direction, can have a significant impact on synaptic function, while a specific research on the genetic basis for nicotine effect on Parkinson's disease, identifies *SV2C* gene as a putative PD-associated gene [47]. Besides, results from previous studies reported the under-expression of other synaptic vesicle proteins, *SV2A* and *SV2B*, consistently with our data (see Table 5).

Other neuronal function-related genes were also retrieved by the single gene level analysis (Table 3), confirming other previous published expression data (Table 5 and S6 Table). In particular, *NPTX2* gene whose up-regulation in Parkinsonian SN was established in single microarray analysis [14, 15, 29], and validated by experiments indicating a localization for NPTX2 protein in Lewy bodies, Lewy neurites and some glial cells [48].

*CBLN1* gene was instead reported under-expressed across microarray studies including our TRAM analysis [13, 29, 30]. It encodes a cerebellum-specific precursor protein, precerebellin, with similarity to the globular (non-collagen-like) domain of complement component C1qB and seems to be a candidate for homeostatic regulation of synapse formation and maintenance [49].

At single gene level, between the twenty most over-expressed genes in PD brain tissue vs. controls, we observed the presence of two alpha-defensin genes located on chromosome 8 (Table 3): *DEFA3*, with the highest expression ratio (4.93) and *DEFA1*, with a 2-fold higher expression in PD pathological tissue. Again, defensins are a family of antimicrobial and cytotoxic peptides thought to be involved in host defense, confirming the possible microglial activity in response to the inflammatory status typical of the Parkinson's disease. To date, however, investigations conducted on alpha-defensins showed their involvement in inflammation typical of several diseases, such as AD [50] or diabetes [51].

Conversely, the meta-analysis conducted on microarray data from LMD DA neurons, has evidenced only few chromosome segments and loci directly related to metabolic pathways which are known to be involved in PD (e.g. immune response, protein ubiquitination, apoptotic process). Instead, our research results have indicated the prevalence of neuronal function genes, transcription factor and regulatory elements, as differentially expressed (Tables 2 and 4).

It is noteworthy that the most over-expressed segment contains the ncRNA transcript, *MLK7-AS1* (2q31-q32) and that between the twenty most variable genes in single gene level analysis, several non-coding transcripts and not-yet-characterized EST clusters have been found, including LINC00520, with an almost ten-fold higher expression in PD patient cells.

Long non-coding RNAs (lncRNAs) are well studied among the thousands non-coding eukaryotic RNAs that have been discovered so far. Their cellular action mechanisms are still largely unknown [52], even if, accumulating evidence suggests that in the nervous system, lncRNA functions may regulate brain evolution and neural development [53], while other results suggest their involvement in neurodegenerative diseases, and specifically PD [54].

Another putative novel regulation sequence is miR-622, indicated by TRAM analysis as under-expressed in DA neurons of PD patients. MicroRNAs (miRNAs) are endogenous, small non- coding RNAs that regulate gene expression by antisense complementarity to the 3'-UTR region of specific mRNAs [55]. It is well known that miRNAs may regulate diverse biological processes such as cell proliferation, apoptosis, stress resistance, stem cell maintenance and cell identity [56]. Recent studies show that miR-622 is associated with tumor metastatic capability in gastric cancers [57] and can suppress glioma invasion and migration by directly targeting activating transcription factor *ATF2* [58], but no data have been released about a miR-622 neuromodulation activity. Our result could suggest a role of miRNAs in the maintenance of dopamine neurons, consistently with a previous study where the authors investigated the role of several miRNAs in the terminal differentiation, function, and survival of mammalian midbrain dopaminergic neurons [59]. The authors identified miR-133b as specifically expressed in DA neurons and reduced in midbrain tissue of PD patients, establishing that it regulates the cells maturation and function, within a negative feedback circuit that includes the paired-like homeodomain transcription factor Pitx3 [59]. Besides, the pro-inflammatory and suppressive role of the most studied neuroimmune miRNAs, miR-155 and miR-146a, has been recently reviewed together with other miRNAs implicated in the pathophysiology of acute and chronic CNS diseases [60]. For this reason, it could be interesting to further investigate miR-622 for a possible similar role in post-transcriptional regulation.

By contrast to SN expression profile, characteristic of neuroinflammation and neurodegeneration, DA neurons expression profile underlies their ability to survive during these chronic processes in Parkinson's disease.

In this context, several studies show that the adaptive stress responses stimulated in various human neurodegenerative diseases, including PD, can confer resistance to a subsequent neurotoxic challenge [61].

In particular, amongst the most over-expressed genes in patients DA neurons, we can note some known genes involved in essential neuronal functions and survival, as the maintenance of cytoskeleton integrity (*TRIM54* and *GAS2L3*) and the neuronal plasticity (*SHISA7*), known to be damaged processes in PD [62, 63].

Contrariwise, between the most under-expressed transcript in patients we can point out *ALDH1A1*, specifically expressed in normal DA neurons and consequently under-represented in PD patients, as shown in several expression studies (see Table 5 and S6 Table); similarly, *AGTR1* gene whose trend is consistent with previous evidence showing that the total cellular AGTR1 levels are drastically reduced in surviving dopamine neurons of PD patients [64].

Overall, a general under-expression profile of the DA neuron associated genes in PD substantia nigra is confirmed also by TRAM analysis (e.g.: *TH*, *SLC6A3*, *DDC*, *EN1*, see S6 Table), compatible with the neuron loss, suggesting that the over-expressed genes could act as moderator of the under-expression of specific genes related to PD and thus contribute to a neurodegenerative-resistant phenotype.

A peculiar result has emerged in PD neurons analysis (Table 2, DA ONLY), as results indicate an almost two-fold higher over-expression of a segment on chromosome 11 (11p15.5). The same locus was already investigated in a previous linkage study of juvenile parkinsonism, even if a specific linkage was excluded in the 10 affected individuals considered [65].

The region contains some members of *MUC* gene family and the single gene level evaluation has also indicated two mucin gene loci as the most over-expressed in DA neurons of Parkinson affected (Table 3). It is well known that mucins are co-secreted with trefoil factor family (TFF)-peptides in a large number of human mucous epithelia [66].

TFF peptides are typical secretory products of a variety of mucin-producing epithelial cells, constituents of mucus gels with a demonstrated anti-apoptotic effects, and a probable modulation in inflammatory processes. The protective effect may operate by organising the mucin layer which protects the mucosa from damage [67]. Interestingly, TFF peptides have also been found widely expressed in rat and mouse nervous central system and in minor amounts in the human brain [66].

Furthermore, Belovari et al. [68], has recently investigated the presence of TFF1 and TFF3 in the nervous system of developing mouse embryos, suggesting their probable involvement in complex processes of nervous system development and differentiation, and brain plasticity.

Notwithstanding this, further studies are necessary to confirm the up-regulation observed in PD patients, as to date very few data are available about MUC genes expression in brain.

In conclusion, this study offers a new approach for the regional analysis of gene expression in Parkinson's disease, by combining multiple data sets from independent studies.

The results of our integrated research globally confirm the deregulation of genes involved in general key cellular functions (mitochondrial energy metabolism, protein degradation, synaptic function) as well as survival mechanisms (immune system processes, response to stimulus) and provide new insights about loci not yet associated with the disease. Further studies are needed to investigate the detailed roles of some of the coding genes and ncRNA resulted in this study.

## Supporting Information

S1 Table Samples selected for the meta-analysis of gene expression profiles of PD patients vs. healthy controls. All Sample and Platforms IDs are related to GEO database. Age at death: patient/control age in years; PMI (hours): post mortem interval before freezing (the time is indicated in hours); CTR/PD: control/Parkinson's disease patient sample; Source: brain tissue and method of extraction; SN: substantia nigra whole tissue; DA: dopaminergic neurons from laser microdissection; Array-Platform-Title: type of array platform used in the analysis; Value type: normalization method; Platform and Sample rows: platform and sample spots number; References and GEO experiment reference. * The serie is not deposited in GEO database [16]. N/A: not available.
(XLS)

S2 Table List of 36,446 TRAM mapped loci for which an expression value A/B was calculated (PD patient SN samples vs. control SN samples). Loci are sorted in descending order. Gene Name: official gene symbol as indicated in Gene database; Chr: chromosome; Data points: number of spots associated to an expression value for the locus; Expression A or B: gene expression mean value of all data available for a locus; Expression A/B: gene expression ratio of value A/value B; SD: standard deviation of the expression value indicated as percentage of the mean. N/A: not available in the Gene database (http://www.ncbi.nlm.nih.gov/gene) when the analysis was performed.
(XLS)

S3 Table Map mode analysis at single gene level of pool A (PD patient SN whole tissue) vs. pool B (control SN whole tissue). The 11,775 resulting loci are sorted in descending order of expression ratio (A/B). Gene Name: official gene symbol as indicated in Gene database; Chr: chromosome; Location: segment cytoband; Segment Start/End: chromosomal coordinates for each segment; Expression A/B: gene expression ratio as mean value of all data available for a locus in pool A or pool B; q: p-value corrected for FDR (False Discovery Rate) of the segment; N/A: location not available in the Gene database (http://www.ncbi.nlm.nih.gov/gene) when the analysis was performed.
(XLS)

S4 Table List of 25,795 TRAM mapped loci for which an expression value C/D was calculated (PD patient DA neuron vs. control DA neuron). Loci are sorted in descending order. Gene Name: official gene symbol as indicated in Gene database; Chr: chromosome; Data points number of spots associated to an expression value for the locus; Expression C or D: gene expression mean value of all data point available for a locus; Expression C/D: gene expression ratio of value C/value D; SD: standard deviation of the expression value indicated as percentage of the mean. N/A: not available in the Gene database (http://www.ncbi.nlm.nih.gov/gene) when the analysis was performed.
(XLS)

S5 Table Map mode analysis at single gene level of pool C (PD patient DA neuron) vs. pool D (control DA neuron). The 7,342 resulting loci are sorted in descending order of expression ratio (C/D). Gene Name: official gene symbol as indicated in Gene database; Chr: chromosome; Location: segment cytoband; Segment Start/End: chromosomal coordinates for each segment; Expression C/D: gene expression ratio as mean value of all data available for a locus in pool C or pool D; Q: p-value corrected for FDR (False Discovery Rate) of the segment; N/A: location not available in the Gene database (http://www.ncbi.nlm.nih.gov/gene) when the analysis was performed.
(XLS)

S6 Table Comparison of TRAM analysis results with the main previously published data. The known genes confirmed in previous single studies are reported (see references indicated). A general trend of over/under-expression was observed for all the considered genes, except for the values in grey boxes. Chr: chromosome; A/B (SN) and C/D (DA): expression ratio of value A/value B (SN ONLY) and value C/value D (DA ONLY) resulted from TRAM analysis (see respectively, S2 and S4 Tables). In bold: expression ratio values statistically significative in single gene level TRAM analysis, q value<0.05 (see respectively, S3 and S5 Tables); GO term Process: description and accession number of the main biological process associated to the gene according to Gene Ontology Consortium.
(DOC)

S7 Table PRISMA checklist. (DOC)
