# Supplementary material for: Meta-Analysis of Parkinson's Disease Transcriptome Data Using TRAM Software: Whole Substantia Nigra Tissue and Single Dopamine Neuron Differential Gene Expression
Source: PLoS One. 2016 Sep 9;11(9):e0161567. doi: 10.1371/journal.pone.0161567 (PMC5017670; doi:10.1371/journal.pone.0161567)
Supplement: S6 Table — The known genes confirmed in previous single studies are reported (see references indicated). A general trend of over/under-expression was observed for all the considered genes, except for the values in grey boxes. Chr: chromosome; A/B (SN) and C/D (DA): expression ratio of value A/value B (SN ONLY) and value C/value D (DA ONLY) resulted from TRAM analysis (see respectively, S2 and S4 Tables). In bold: expression ratio values statistically significative in single gene level TRAM analysis, q value<0.05 (see respectively, S3 and S5 Tables); GO term Process: description and accession number of the main biological process associated to the gene according to Gene Ontology Consortium. (DOC) [file pone.0161567.s006.doc]

**S6 Table: Comparison of TRAM analysis results with the main previously published data.**

| **Genes** | **Chr** | **A/B (SN)** | **C/D (DA)** | **References** | **GO term Process** |
| --- | --- | --- | --- | --- | --- |
| *ACTN4* | chr19 | **1.37** | 1.26 | [29] | positive regulation of cellular component movement (GO:0051272) |
| *ACTR3* | chr2 | 0.93 | **0.58** | [12] | movement of cell or subcellular component (GO:0006928) |
| *AGTR1* | chr3 | **0.46** | **0.34** | [13, 29, 30] | signal transduction (GO:0007165) |
| *ALDH1A1* | chr9 | **0.41** | **0.21** | [12, 13, 29, 30] | cellular aldehyde metabolic process (GO:0006081) |
| *ANK1* | chr8 | **0.43** | 0.71 | [13, 19, 29, 30] | cytoskeleton organization (GO:0007010) |
| *ATP5A1* | chr18 | 0.81 | **0.54** | [16] | mitochondrial ATP synthesis coupled proton transport (GO:0042776) |
| *ATP5C1* | chr10 | 0.89 | **0.50** | [19] | mitochondrial ATP synthesis coupled proton transport (GO:0042776) |
| *ATP5J* | chr21 | 0.93 | **0.48** | [16, 19] | mitochondrial ATP synthesis coupled proton transport (GO:0042776) |
| *ATP5L* | chr11 | 0.99 | **0.59** | [16, 19] | mitochondrial ATP synthesis coupled proton transport (GO:0042776) |
| *ATP6V0D1* | chr16 | 0.87 | **0.51** | [19] | proton transport (GO:0015992) |
| *ATP6V1B2* | chr8 | 0.76 | **0.60** | [19] | proton transport (GO:0015992) |
| *ATP6V1D* | chr14 | 0.89 | **0.57** | [19, 30] | proton transport (GO:0015992) |
| *BEX1* | chrX | 0.70 | **0.41** | [14, 19, 29, 30] | up regulation of transcription factor (GO:0045944) |
| *BOK* | chr2 | 2.10 | **1.2** | [14] | apoptotic process (GO:0006915) |
| *CACNB3* | chr12 | 0.57 | **0.60** | [16] | generation of L-type calcium current (GO:0061577) |
| *CADPS* | chr3 | **0.39** | 0.77 | [16] | exocytosis (GO:0006887) |
| *CBLN1* | chr16 | **0.52** | 0.70 | [13, 29, 30] | synaptic transmission (GO:0007268) |
| *CBR1* | chr21 | **1.36** | 1.12 | [12] | oxidation-reduction process (GO:0055114) |
| *CDH10* | chr5 | **0.51** | 1.23 | [19] | single organismal cell-cell adhesion (GO:0016337) |
| *CHCHD2* | chr7 | 0.97 | **0.66** | [12] | up regulation of transcription factor (GO:0045944) |
| *CLDN11* | chr3 | **1,39** | **0.55** | [29] | calcium-independent cell adhesion molecule activity (GO:0016338) |
| *COL16A1* | chr1 | **1.48** | 1.43 | [30] | cell adhesion (GO:0007155) |
| *COX6B1* | chr19 | 1.06 | **0.55** | [19] | mitochondrial electron transport, cytochrome c to oxygen (GO:0006123) |
| *COX6C* | chr8 | 1.02 | **0.51** | [16, 19] | mitochondrial electron transport, cytochrome c to oxygen (GO:0006123) |
| *CRYAB* | chr11 | **1.58** | 1.79 | [30] | protein folding (GO:0006457) |
| *CXCR4* | chr2 | **1.5** | **2.42** | [13] | G-protein coupled receptor signaling pathway (GO:0007186) |
| *DDC* | chr7 | **0.35** | 0.66 | [19] | dopamine biosynthetic process (GO:0042416) |
| *DDIT4* | chr10 | **1.47** | **2.31** | [30] | negative regulation of TOR signaling (GO:0032007) |
| *DNAJB2* | chr2 | **1.38** | 0.77 | [19] | protein folding (GO:0006457) |
| *DNM1* | chr9 | 0.73 | **0.60** | [16, 19] | endocytosis (GO:0006897) |
| *DNPEP* | chr2 | **0.49** | 0.93 | [16] | peptide metabolic process (GO:0006518) |
| *DYNC1I1* | chr7 | 0.68 | **0.53** | [16, 19] | vesicle transport along microtubule (GO:0047496) |
| *DYNLL1* | chr12 | 1.10 | **0.57** | [16] | actin cytoskeleton organization (GO:0030036) |
| *DYNLL1* | chr12 | 1.10 | **0.57** | [16] | actin cytoskeleton organization (GO:0030036) |
| *EGLN1* | chr1 | **1.33** | 0.99 | [12] | response to hypoxia (GO:0001666) |
| *EN1* | chr2 | **0.52** | 0.61 | [19] | dopaminergic neuron differentiation (GO:0071542) |
| *FAS* | chr10 | **0.50** | 1.21 | [16] | apoptotic process (GO:0006915) |
| *FGF13* | chrX | **0.40** | 0.69 | [14, 19, 30] | signal transduction (GO:0007165) |
| *GABRB1* | chr4 | **0.52** | 0.72 | [16, 29] | signal transduction (GO:0007165) |
| *GBE1* | chr3 | **0.40** | **0.48** | [12] | glycogen metabolic process (GO:0005977) |
| *GLS* | chr2 | **0.45** | 0.72 | [16] | glutamate biosynthetic process (GO:0006537) |
| *GNAI1* | chr7 | **1.46** | **0.55** | [12] | G-protein coupled receptor signaling pathway (GO:0007186) |
| *GTPBP4* | chr10 | **0.44** | 0.71 | [16] | regulation of CDK activity (GO:0000079) |
| *GTPBP4* | chr10 | **0.44** | 0.71 | [16] | regulation of cyclin-dependent protein serine/threonine kinase activity (GO:0000079) |
| *HIPK2* | chr7 | **1.36** | 1.19 | [30] | transcription, DNA-templated (GO:0006351) |
| *HLTF* | chr3 | 0.82 | **0.58** | [12] | regulation of transcription, DNA-templated (GO:0006355) |
| *HSPB1* | chr7 | **1.63** | 2.08 | [14, 30] | intracellular signal transduction (GO:0035556) |
| *IMMT* | chr2 | 0.75 | **0.51** | [16] | cristae formation (GO:0042407) |
| *JMJD6* | chr17 | **1.63** | 1.22 | [14, 30] | histone demethylation (GO:0016577) |
| *JUP* | chr17 | **1.33** | 1.10 | [29] | single organismal cell-cell adhesion (GO:0016337) |
| *KCMF1* | chr2 | 0.84 | **0.59** | [16] | metabolic process (GO:0008152) |
| *KCNJ6* | chr21 | **0.45** | 0.85 | [16] | potassium ion transport (GO:0006813) |
| *KIF5B* | chr10 | **1.33** | 0.90 | [16] | microtubule base movement (GO:0007018) |
| *KLHL21* | chr1 | **1.31** | 0.93 | [30] | protein ubiquitination (GO:0016567) |
| *LSS* | chr21 | **1.34** | 0.87 | [30] | steroid biosynthetic process (GO:0006694) |
| *MAN2A1* | chr5 | **1.49** | 0.65 | [30] | N-glycan processing (GO:0006491) |
| *MKNK2* | chr19 | **1.5** | 1.15 | [14, 30] | regulation of translation (GO:0006417) |
| *MRPL15* | chr8 | **0.39** | 0.70 | [16] | mitochondrial translation (GO:0032543) |
| *MRPL3* | chr3 | 0.76 | **0.58** | [16] | mitochondrial translation (GO:0032543) |
| *MYOM1* | chr18 | 0.99 | **0.36** | [12] | mitophagy (GO:0000422) |
| *NDUFA1* | chrX | 0.97 | **0.51** | [16] | complex I (NADH to ubiquinone) (GO:0006120) |
| *NDUFB2* | chr7 | 0.88 | **0.44** | [16, 19] | complex I (NADH to ubiquinone) (GO:0006120) |
| *NDUFS1* | chr2 | **0.72** | 0.68 | [30] | complex I (NADH to ubiquinone) (GO:0006120) |
| *NPTX2* | chr7 | **2.13** | 1.42 | [15, 29] | synaptic transmission (GO:0007268) |
| *ODC1* | chr2 | 0.84 | **0.57** | [16] | polyamine metabolic process (GO:0006595) |
| *P2RX7* | chr12 | **1.45** | 2.04 | [29] | positive regulation of ion transmembrane transport (GO:0034767) |
| *PLCL1* | chr2 | **1.35** | 0.85 | [12] | intracellular signal transduction (GO:0035556) |
| *PPA1* | chr10 | 1.22 | **0.56** | [12] | diphosphate metabolic process (GO:0071344) |
| *PSMB4* | chr1 | 0.88 | **0.55** | [16] | proteasome-mediated ubiquitin-dependent protein catabolic process (GO:0043161) |
| *REEP1* | chr2 | **0.47** | **0,58** | [30] | protein insertion into membrane (GO:0051205) |
| *RGS4* | chr1 | **0.46** | **0.54** | [14, 30] | regulation of GPCR protein signalling pathway (GO:0008277) |
| *RIMS1* | chr6 | **0.52** | 0.86 | [16] | synaptic vescicle exocytosis (GO:0016079) |
| *SASH1* | chr6 | **1.38** | 0.87 | [30] | protein polyubiquitination (GO:0000209) |
| *SCN3B* | chr11 | **0.53** | 0.81 | [16] | regulation of sodium ion transmembrane transporter activity (GO:2000649) |
| *SCRN1* | chr7 | 0.92 | **0.60** | [16] | exocytosis (GO:0006887) |
| *SGK1* | chr6 | **1.58** | 0.81 | [29] | positive regulation of transporter activity (GO:0032411) |
| *SLC24A2* | chr9 | **1.54** | 1.61 | [16] | ion transmembrane transport (GO:0034220) |
| *SLC31A2* | chr9 | 1.19 | **0.48** | [12] | copper ion transport (GO:0006825) |
| *SLC6A3* | chr5 | **0.43** | 0.91 | [19] | dopamine transport (GO:0015872) |
| *SLCO4A1* | chr20 | **1.70** | 1.23 | [30] | sodium-independent organic anion transport (GO:0043252) |
| *SRD5A1* | chr5 | **0.44** | 0.76 | [30] | androgen biosynthetic process (GO:0006702) |
| *STAT2* | chr12 | **1.41** | 0.96 | [29] | regulation of transcription from RNA polymerase II promoter ( GO:0006357) |
| *SUMO3* | chr21 | 0.87 | **0.58** | [16] | protein sumoylation (GO:0016925) |
| *SV2B* | chr15 | **0.45** | 0.82 | [14, 16, 30] | neurotransmitter transport (GO:0006836) |
| *SV2C* | chr5 | **0.31** | **0.60** | [30] | neurotransmitter transport (GO:0006836) |
| *SYT1* | chr12 | 0.54 | **0.58** | [14, 16, 19, 30] | synaptic transmission (GO:0007268) |
| *TAC1* | chr7 | **0.32** | 1.72 | [30] | innate immune response (GO:0045087) |
| *TF* | chr3 | **1.33** | 0.80 | [14, 15, 30] | iron ion homeostasis (GO:0055072) |
| *TH* | chr7 | **0.48** | 1.05 | [29] | dopamine biosynthetic process (GO:0042416) |
| *TMEFF1* | chr9 | **0.49** | **0.60** | [12] | multicellular organism development (GO:0007275) |
| *TOMM20* | chr1 | 0.90 | **0.60** | [16] | protein targeting to mitochondrion (GO:0006626) |
| *TUBB2B* | chr6 | 0.88 | **0.58** | [16] | microtubule-based process (GO:0007017) |
| *TUBB3* | chr16 | 0.61 | **0.57** | [16] | microtubule-based process (GO:0007017) |
| *TUBB4B* | chr9 | **1.30** | 0.69 | [16] | microtubule-based process (GO:0007017) |
| *TUBD1* | chr17 | **1.29** | 1.45 | [15, 16] | microtubule-based process (GO:0007017) |
| *UQCRC2* | chr16 | 0.66 | **0.55** | [12, 16, 19] | mitochondrial electron transport, ubiquinol to cytochrome c (GO:0006122) |
| *VAV3* | chr1 | 0.61 | **0.57** | [14] | positive regulation of GTPase activity (GO:0043547) |
| *VDAC3* | chr8 | 0.85 | **0.53** | [19] | regulation of anion transmembrane transport (GO:1903959) |
| *VEGFA* | chr6 | **1.30** | 0.77 | [12] | cellular response to vascular endothelial growth factor stimulus (GO:0035924) |
| *YWHAE* | chr17 | **1.43** | 0.96 | [30] | intracellular signal transduction (GO:0035556) |
| *ZBTB16* | chr11 | **1.45** | 1.63 | [29, 30] | transcription, DNA-templated (GO:0006351) |
| *ZNF160* | chr19 | 1.22 | **2.39** | [30] | regulation of transcription, DNA-templated (GO:0006355) |
